# Effect of Pro-kin visual feedback balance training on balance function of individuals with early Parkinson's disease: a randomized controlled pilot trial

**DOI:** 10.4314/ahs.v23i2.67

**Published:** 2023-06

**Authors:** Tingting Han, Qian Liu, Yaguang Hu, Yuxia Wang, Kangying Xue

**Affiliations:** 1 Sino-French Department of Neurological Rehabilitation, Gansu Provincial Hospital, Lanzhou, Gansu, China; 2 Cerebrovascular Disease Center, Gansu Provincial Hospital, Lanzhou, Gansu, China; 3 Department of Pharmacy, Gansu Provincial Maternity and Child-care Hospital, Lanzhou, Gansu, China; 4 Department of Respiratory Medicine, Gansu Provincial Hospital, Lanzhou, Gansu, China; 5 Vaccination Hub, Monash Health, Melbourne, Victoria, Australia

**Keywords:** Balance, parkinson, rehabilitation, visual feedback

## Abstract

**Background:**

Parkinson's disease (PD) is the second most common neurodegenerative disease. Patients often present with balance dysfunction. Several studies have applied visual feedback training to stroke patients and demonstrated significant improvement. However, the application of visual feedback balance training in PD patients has not been reported.

**Objective:**

To observe the effects of visual feedback balance training combined with conventional rehabilitation training on the balance function of patients with early PD.

**Methods:**

Fifty patients with early PD were randomly divided into control group and observation group. The control group received conventional rehabilitation training, including body position transfer, weight shifting, movement in all directions and gait training. The observation group were added with visual feedback balance training on the basis of the training above. All patients were trained 5 times per week for 4 weeks. Berg Balance Scale (BBS), Time Up-and-Go test (TUG) and Pro-Kin balance training instrument were used to evaluate the balance function of patients before and after treatment, and the balance function were compared between the two groups.

**Results:**

The BBS and TUG scores of the observation group and the control group were improved significantly (P<0.01), and the BBS and TUG scores of the observation group were improved more obviously than control group (P<0.01). The length and area of eye open and closed condition in the observation group and the control group were significantly reduced compared with those before training (P<0.01), and the degree of reduction in the observation group was more obvious (P<0.01). The length and area of the observation group and the control group before and after training when eye open were smaller than those when eye closed (P<0.01).

**Conclusion:**

The conventional rehabilitation therapy can improve the balance function of PD patients, but the combination of visual feedback balance training and conventional rehabilitation therapy can improve the balance function more significantly.

## Introduction

Parkinson's disease (PD) is a chronic progressive neurodegenerative disease in the elderly, which is characterized by prominent death of dopaminergic neurons in the substantia nigra pars compacta and wide spread presence of alpha synuclein, an intracellular protein^1^. The disease is the second most common neurodegenerative disease, affecting approximately 7 million people worldwide^2^. PD is the fastest growing disease among neurological disorders which are now the leading cause of disability. The number of individuals with PD is poised for exponential growth because the world's population is aging^3^. The symptoms of PD include tremor, bradykinesia, rigidity, balance dysfunction and non-motor disorders like sleep problems^4^.

Falls are frequent and recurrent events in individuals with PD, with 45-68% of people with PD falling annually and two-thirds of them experiencing more than once^5^. Falls cause many problems such as hip fracture, pain, loss of independence and activity limitations. The risk factors associated with falls in PD include balance dysfunction, freezing gait, cognitive impairment, reduced leg muscle strength, fear, depression^6^. Among these factors, balance dysfunction leads to increased body swing, poor coordination and posture instability. It becomes more serious with PD progressing. As the result, balance dysfunction has been regarded as one of the independent risk factors of falls^7^.

Non-pharmacological treatment, such as exercise and physical therapy, demonstrates many benefits for individuals with PD including improvements in stability and balance, gait and independence^8^. Study has shown that long-term rehabilitation training can improve the motor ability and balance function of individuals with PD^9^. However, conventional rehabilitation training, such as muscle strengthening, center of gravity shift and stretching, is difficult to arouse interest and maintain long-term training. In addition, conventional rehabilitation treatment is difficult to evaluate the training result in real time, which makes it difficult for the therapist to make further treatment plans.

The Pro-kin system is designed for balance training, which provides visual feedback of center of gravity in real time, making it possible to adjust balance according to system feedback.

The Pro-kin training system has been reported for the balance function treatment of individuals with white matter lesions^10^, cerebral small vessel disease^11^. The two studies have demonstrated that it is a feasible method to improve balance function. However, it has never been reported in the treatment of PD patients. The purpose of this study was to investigate the effect of Pro-kin visual feedback training system on balance function of individuals with early PD.

## Materials and methods

Patients who were diagnosed with PD by a doctor from June 2020 to October 2021 were recruited from the Sino-French Department of Neurological Rehabilitation of Gansu Provincial Hospital. Patients should have no less than two of the four classic motor symptoms of PD, including resting tremor, rigidity, bradykinesia and asymmetric onset^12^. The inclusion criteria were: (1) Hoehn and Yahr stage 1 to 2, (2) 45 to 70 years, (3) the course of disease is within 3 years, (4) stable medication, (5) ability to understand instructions from therapist or instrument and perform required actions^13^. The exclusion criteria were: (1) secondary Parkinson's syndrome and Parkinson's syndrome caused by infection, cerebral arteriosclerosis and trauma. (2) inability to cooperate with treatment because of consciousness disorder or severe mental disorder, (3) unable to perform balance training because of lower limb fracture, severe pain or tumor. (4) prior history of epilepsy.

Fifty patients were recruited into this study and randomly divided into two groups: the control group and the intervention group. Randomization was performed by a doctor who was not involved in the study. All participants signed informed consents.

The study was a single blind randomized controlled trial in which the evaluator was unaware of the treatment. Pretest and post-test designs were used to evaluate the effectiveness of the training. Participants were randomly assigned to the control group or the observation group by a physician who was not involved in the study. An opaque sealed envelope was selected from 50 sealed envelopes containing group assignment.

All subjects were assessed by the Berg Balance Scale (BBS), the Time Up-and-Go Test (TUG) and the built-in assessment procedures of the Pro-Kin balance training system before the training. span style=“font-family: ‘Times New Roman’; color:#0070c0; background-color:#ffffff”>The specific evaluation methods are as follows: First, subjects were asked to complete 14 items in turn according to the BBS scale, including sitting independently, standing with eyes closed, standing on one leg, walking up and down stairs, turning around for a week and so on14. Each item is rated from 0 to 4 points according to the degree of completion. The higher the score is, the better the completion is. The scores of all items are added up to obtain the total score of the BBS test. Then, the subjects were asked to conduct TUG test15. The subjects were asked to stand up from the chair, walk 3 meters to the indicating line, then turn around and sit on the chair. The therapist recorded the time spent. The average time of three tests were the result of TUG test. Finally, the built-in evaluation program of Pro-kin Visual Feedback Balance Training System (PK254, Tec nobody, Italy) was used for testing balance function. Turn on the computer and click the “Static stability Test” option on the screen. Therapist asked the subject to stand on the test table with their eyes looking straight ahead and tell the subject to hold as still as possible for 30 seconds. Then, therapist asked the subject to close eyes and hold as still as possible for 30 seconds. The system automatically recorded the movement of the center of gravity and depicted the curve of change. The total length of the trajectory generated by the center of gravity motion was called the trajectory length, and the area enclosed by the trajectory generated by the center of gravity motion was called the area of the moving ellipse. The smaller the value of these two parameters, the better the balance function. Each patient was tested twice.

After the evaluation, all subjects received rehabilitation training. All participants were given conventional balance training, including body position transfer, weight shifting, movement in all directions, standing on one leg, touching objects, turning around and throwing a ball. The training was conducted 30 minutes per session, 5 times a week, for 4 weeks.

The observation group was added with visual feedback balance training in addition to conventional balance training, for 20 minutes per session, 5 times a week for 4 weeks. The Pro-kin visual feedback balance training system was used. The subjects stood on the training board. The central axis of the feet was located on the A1 and A5 lines of the balance platform. The highest points of the bilateral arches were located on the A3 and A7 lines of the balance platform respectively. The Angle between the feet was 60 degrees. The training method was as follows: four fixed locks were placed under the training board, then the subjects stood on the board and looked at the computer screen. By clicking on the “stability limit” module on the computer, the subjects controlled the slight movement of the training table with their feet to control the cursor on the screen to a specified position. Next, the therapist removed the fixed lock under the training board and adjusted the resistance value to the system-recommended 5. Then the subject placed one foot on the training board and the other foot on the ground, keeping the upper body still. The subject looked at the computer screen and controlled the large amplitude movement of the balance board through the ankle joint according to the cursor indication on the screen, making the cursor on the screen reach the specified position.

### Statistics

SPSS 24.0 software were used to analyse the data. Descriptive statistics were used for all outcome variables. The means and standard deviations were reported, which were expressed as-x±s. independent t-test was used for between-group comparison, and paired t-test was used for within-group comparison. The quantitative data were compared by chi-square test. P <0.01 was considered statistically significant.

## Results

One case fell off in the observation group and one case fell off in the control group after training, which was due to unwillingness to continue the training. There were no difference between groups in terms of gender, age, duration of disease and the Hoehn and Yahr scale grades(Table 1).

**Table 1:** Demographic data of the participants

	Observation group(n=24)	Control group(n=24)	P
Gender(n)			
Men	12	11	0.773
Women	12	13	
Age(years)	58.17±5.36	56.25±4.82	0.199
Height(cm)	1.66±0.04	1.66±0.03	0.345
Weight(kg)	65.79±2.36	64.54±2.36	0.073
Disease duration(years)	1.71±0.75	1.83±0.70	0.554
Hoehn and Yahr scale(grades)	1.33±0.48	1.38±0.49	0.769

The BBS and TUG scores of the observation group and the control group were improved significantly after training (P<0.01). The BBS and TUG scores of the observation group were improved more obviously than those of the control group (P<0.01) (Table 2).

**Table 2:** BBS and TUG scores before and after treatment

	Intervention group (n=24)	Control group (n=24)	P
BBS (scores)			
Pre-test	43.04±1.52	42.67±1.40	0.379
Post-test	45.96±1.12	50.29±1.85	0.000
P	0.000	0.000	
TUG (seconds)			
Pre-test	15.73±0.88	15.92±0.84	0.444
Post-test	11.73±0.60	13.08±0.58	0.000
P	0.000	0.000	

The length and area of eye open and eye closed condition in the observation group and the control group were significantly reduced compared with those before training (P<0.01), and the degree of reduction in the observation group was more obvious (P<0.01) (Table 3). The length and area of the observation group and the control group before and after training when eye open were all smaller than those when eye closed (P<0.01) (Table 3).

**Table 3:** Evaluation results from Pro-kin balance system

	Observation group (n=24)	Control group (n=24)	P
Eyes open			
Trajectory length(mm)			
Before treatment	701.17±29.21	666.71±130.11	0.212
After treatment	327.54±43.82	411.00±18.35	0.000
P	0.000	0.000	
Ellipse area(mm^2^)			
Before treatment	1231.42±152.27	1185.96±153.42	0.308
After treatment	649.96±31.56	834.88±22.23	0.000
P	0.000	0.000	
Eyes closed			
Trajectory length(mm)			
Before treatment	931.29±71.97	959.42±69.95	0.176
After treatment	443.13±44.76	617.83±14.40	0.000
P	0.000	0.000	
Ellipse area(mm^2^)			
Before treatment	2161.38±155.84	2090.42±149.01	0.114
After treatment	1166.17±83.31	1684.50±52.33	0.000
P	0.000	0.000	

## Discussion

There are many factors leading to abnormal balance function in PD patients. On one hand, the loss of dopamine substantia nigra neurons leads to the decrease in dopamine, which in turn leads to motor dysfunction. On the other hand, decreased activity of the cholinergic system as a result of cholinergic neurons in peduncu lopontine nucleus and basal forebrain leads to balance dysfunction. In addition, abnormal activity in the cerebral cortex, the brain stem network and the midbrain motor area substantia nigra may cause motor dysfunction. Cerebellar lesions can also cause motor abnormalities and balance disorders^16^. Balance dysfunction is not always improved by dopami nergic drugs^17^, and also can interfere with walking^18^. As shown in this study, before treatment the TUG time of the experimental group and the control group was 15.73±0.88 seconds and 15.92±0.84 seconds respectively, which means an increased risk of falls in patients with PD^19^. Studies have shown that improved balance function may reduce falls and disability due to fractures^20^. Drug therapy cannot completely resolve the loss of balance function, so non-drug therapy is necessary. Lei gave virtual reality rehabilitation training to PD patients on the basis of conventional treatment, which showed significant improvement in balance ability^21^. Yang provided rehabilitation training for early PD patients with drugs combined with TaiChi, and the results showed that the balance and motor ability were significantly improved^22^. This study also showed that whether combined with visual feedback balance training or just conventional rehabilitation training, balance function was improved. Studies have shown that drug therapy focuses on improving myotonic and bradykinesia of PD patients, but does not improve balance ability^23^. Strength and balance training can improve muscle strength, coordination and balance function of PD patients.

The conventional rehabilitation treatment for PD patients has been reported, such as endurance training^24^, Nordic walking training^25^, which showed significant improvement in balance function. Now there are more and more scholars combined conventional rehabilitation training with audio-biofeedback training^26^, or resistance training^27^, or rhythmic auditory stimulation with visual stimuli^28^. The methods above all improve balance function, but there is no study about which is better. Some studies have applied visual feedback training to stroke patients and proved that it can improve balance function significantly. However, no visual feedback balance training has been applied to PD rehabilitation treatment^29^.

This study shows that the combination of conventional rehabilitation training and visual feedback balance training can improve the balance ability of early PD patients more significantly. Conventional rehabilitation training focuses on the movement control, center of gravity shift and precision training^30^. PD patients are often unable to complete the movement accurately and co-ordinately due to the presence of tremors, myotonia and other obstacles. By training patients to touch objects, reach for objects and alternate weight bearing, coordination and accuracy of motor completion can be strengthened^31^. However, conventional rehabilitation training cannot distinguish the three sensory systems on which human balance depends. Visual and proprioceptive assistance interferes with training. In addition, conventional rehabilitation training needs to repeat fixed action for many times, while patients are easy to get bored. The data of evaluating the quality of each training cannot be objectively given. The visual feedback balance training has a good human-computer interface, which can develop a gradual training mode for patients and strengthen the proprioception training. Pro-prioception is an efferent activity which senses joint position, joint velocity and muscle tone. It plays an important role in balance maintenance. Visual feedback balance training system is a combination of coordination training, muscle strength training and proprioception training32. Therapists can adjust the balance system according to the situation and change the difficulty of training. The built-in procedure can accurately feel the load change of each point so that patients can gradually and accurately carry out the balance training of load control. In addition, the good human-computer interaction mode greatly improves the interest of patients. Patients can conduct treatment in the game and therapists can make rehabilitation plans according to the system feedback.

## Conclusion

The conventional rehabilitation treatment can improve the balance function of PD patients. This study shows that the combination of visual feedback balance training and conventional rehabilitation treatment can improve the balance function of PD patients more significantly.
